# Preoperative Quantitative MR Tractography Compared with Visual Tract Evaluation in Patients with Neuropathologically Confirmed Gliomas Grades II and III: A Prospective Cohort Study

**DOI:** 10.1155/2016/7671854

**Published:** 2016-04-17

**Authors:** Anna F. Delgado, Markus Nilsson, Francesco Latini, Johanna Mårtensson, Maria Zetterling, Shala G. Berntsson, Irina Alafuzoff, Jimmy Lätt, Elna-Marie Larsson

**Affiliations:** ^1^Department of Surgical Sciences, Radiology, Uppsala University, 75105 Uppsala, Sweden; ^2^Department of Neuroradiology, Karolinska University Hospital, Department of Clinical Neuroscience, Karolinska Institute, 17177 Stockholm, Sweden; ^3^Bioimaging Center, Lund University, 22100 Lund, Sweden; ^4^Department of Neuroscience, Neurosurgery, Uppsala University, 75105 Uppsala, Sweden; ^5^Department of Neuroscience, Neurology, Uppsala University, 75105 Uppsala, Sweden; ^6^Section of Pathology, Uppsala University Hospital and Department of Immunology, Genetics and Pathology, Uppsala University, 75105 Uppsala, Sweden; ^7^MR Department, Centre for Medical Imaging and Physiology, Lund University Hospital, 22185 Lund, Sweden

## Abstract

*Background and Purpose.* Low-grade gliomas show infiltrative growth in white matter tracts. Diffusion tensor tractography can noninvasively assess white matter tracts. The aim was to preoperatively assess tumor growth in white matter tracts using quantitative MR tractography (3T). The hypothesis was that suspected infiltrated tracts would have altered diffusional properties in infiltrated tract segments compared to noninfiltrated tracts.* Materials and Methods.* Forty-eight patients with suspected low-grade glioma were included after written informed consent and underwent preoperative diffusion tensor imaging in this prospective review-board approved study. Major white matter tracts in both hemispheres were tracked, segmented, and visually assessed for tumor involvement in thirty-four patients with gliomas grade II or III (astrocytomas or oligodendrogliomas) on postoperative neuropathological evaluation. Relative fractional anisotropy (rFA) and mean diffusivity (rMD) in tract segments were calculated and compared with visual evaluation and neuropathological diagnosis.* Results.* Tract segment infiltration on visual evaluation was associated with a lower rFA and high rMD in a majority of evaluated tract segments (89% and 78%, resp.). Grade II and grade III gliomas had similar infiltrating behavior.* Conclusion.* Quantitative MR tractography corresponds to visual evaluation of suspected tract infiltration. It may be useful for an objective preoperative evaluation of tract segment involvement.

## 1. Introduction

Gliomas comprise approximately 30% of all CNS tumors and 80% of all malignant brain tumors [1]. In adults, these tumors consist mainly of astrocytomas grades II–IV and oligodendrogliomas grades II-III [2]. In suspected low-grade gliomas, preoperative MRI shows a high signal intensity lesion on T2-FLAIR images, usually without but sometimes with faint/patchy contrast enhancement [3, 4]. Low-grade gliomas are less common than grade IV gliomas, and patients with low-grade gliomas are often younger and have a high quality of life and a longer survival [5]. Survival in patients with glioma increases with the extent of tumor resection at surgery [6, 7]. Total resection of the tumor is hampered by infiltrative growth along white matter tracts [8]. Planning the surgical resection of these tumors is facilitated by knowledge about infiltration of the adjacent tracts. Intraoperative subcortical mapping using direct electrical stimulations under awake surgery is the gold standard to directly assess the functional connectivity of tracts adjacent to the tumor [9]. Retrospective studies have shown increased extent of resection and preserved functions with the help of preoperative physiological MRI, intraoperative neuronavigation, and neurophysiological intraoperative testing [10–12]. Presurgical planning is thus of importance in this group because maximal tumor resection has to be balanced with the preservation of neurological function [13, 14].

Astrocytomas and oligodendrogliomas grade II and grade III have overlapping radiologic features, and on contrast enhanced CT or MRI they can be identified by their lack of typical grade IV features like ring-like contrast enhancement and central necrosis [15, 16]. Gliomas grade II and grade III can be separated on the basis of cytological features, cell density, and mitotic figures on neuropathological assessment. Grade IV gliomas have neuropathological characteristics of irregular vascularization with areas of necrosis [2]. Despite their relatively sharp demarcation on T2-weighted images, low-grade gliomas are known for their infiltrative growth pattern along white matter tracts [17, 18].

Morphological MRI with conventional T1- and T2-weighted images and contrast agent injection provides information about tumor location, the extent of bulk tumor growth, and blood-brain-barrier breakdown and edema, but it does not readily aid in the evaluation of specific white matter tracts. White matter tracts can be displaced, infiltrated, or destructed by tumor growth as evaluated by brain tumor specimen for histopathological evaluation [19–21]. Preoperative noninvasive assessment of white matter structure and glioma growth by diffusion MRI is a promising tool in the presurgical clinical evaluation.

DTI (diffusion tensor imaging) enables the depiction of anisotropic diffusion, which is dependent on white matter fiber orientation [22]. Diffusion tensor tractography (DTT) shows anisotropic diffusion, measured with DTI as color-coded streamlines [23]. DTT depicts white matter fiber orientation and allows visualization of tumor influence on white matter tracts, beyond that of morphological MRI [24]. MD (mean diffusivity) and FA (fractional anisotropy) can be extracted from DTI. MD describes the mean diffusion in tissues and FA describes the directional diffusion in tissues [25]. MD is low in tissue with high cell density and FA is high in tissues with ordered and orientationally coherent microstructure [25]. Previous studies have shown a correlation between lower FA and white matter destruction by tumor. A study by Wang et al. from 2012 demonstrated reduced FA in white matter invaded by tumor cells in a rat glioma model [19]. Reduced FA has also been correlated with increased cell density in gliomas in humans [26].

Earlier studies have evaluated MD and FA in single regions of interest in gliomas showing differences in diffusion between glioma grades [27, 28]. Retrospective studies have evaluated glioma infiltration in tracts by DTT showing the infiltrative growth pattern in gliomas [29]. Quantitative DTT has been reported to correlate with symptoms in glioma patients [30]. The value of DTT in pre- and perioperative imaging has been studied [11, 12, 31–33]. Although a promising method, DTT has several limitations and clinical implications still need further validation [34].

A well-conducted previous study has evaluated and validated DTT in a cohort of normal individuals [35]. Based on these earlier DTT descriptions, the aim of this study was to compare quantitative analysis of MD and FA in tract segments of the major associative, projection, and commissural bundles with visual tract evaluation in patients with nonnecrotic suspected low-grade gliomas and subsequent confirmed neuropathological diagnosis of glioma grade II or grade III. Taking into account the variation in diffusion along the same tract, we systematically divided each bundle into segments using anatomical landmarks [36–38]. To reduce interindividual differences based on, for example, age and degenerative changes in the white matter between patients we normalized the ipsilateral tract segments to the contralateral tract. The resulting DTT findings were compared with visual tract evaluation and neuropathological tumor diagnosis. The hypothesis was that suspected infiltrated tracts would have altered diffusional properties in infiltrated tract segments compared to noninfiltrated tracts.

## 2. Materials and Methods

Fifty consecutive patients were asked to participate in this prospective study, approved by the regional ethical review board in Uppsala (2010/015), which was undertaken from May 2010 to February 2014. Two patients declined participation in the study and were not included. All patients (*n* = 48) gave written informed consent before taking part in the study. Patients eligible for inclusion were those referred to the neurosurgical department with clinical and radiological suspicion of a low-grade glioma. Inclusion criteria were tumors appearing low-grade on morphological MRI with no contrast enhancement or only patchy and faint contrast enhancement [4]. Exclusion criteria were the appearance of high-grade glioma features on morphological MRI with ring-like contrast enhancement and central necrosis. Previously published results from parts of this cohort have evaluated methionine-PET, perfusion, and diffusion characteristics [39, 40]. No study evaluating diffusion tensor tractography in this cohort has been reported. Included patients underwent presurgical radiological imaging, including morphological and physiological MRI with DTI, except for one patient (patient number 3) who had a biopsy 6 months prior to the study MRI. Patients eligible for tractographic evaluation and statistical analysis were those with a neuropathological report following surgery or biopsy of an astrocytoma or oligodendroglioma grade II or III. Exclusion criteria for statistical analysis were the following: no neuropathological diagnosis or diagnosis other than astrocytoma or oligodendroglioma grade II or III on neuropathological assessment.

*MRI*. MRI was performed on a 3 T scanner (Philips Achieva, Philips Medical Systems, Best, Netherlands) and a 32-channel head coil.

### 2.1. Morphological MRI

Morphological sequences included axial T2-FLAIR (TR/TE 11,000/125 ms; 90-degree flip angle; 512 × 512 matrix; and 0.45 × 0.45 × 6.00 mm^3^ voxel size), axial T1-weighted spin echo sequences (TR/TE 600/10 ms; 70-degree flip angle; 512 × 512 matrix; and 0.45 × 0.45 × 5.00 mm^3^ voxel size) before and after intravenous administration of gadobutrol (Gadovist®, Bayer Schering Pharma, Wedding, Berlin, Germany), sagittal T1-weighted 3D turbo field echo (TFE) after contrast agent injection (TR/TE: 8.1/3.7 ms; voxel size 1 × 1 × 1 mm), and sagittal T2-weighted turbo spin echo (SE) (TR/TE = 3,000/80 ms; slice thickness = 4 mm; and slice gap = 0.8 mm). Sequences acquired but not assessed in this study were axial T2-weighted turbo spin echo (SE), coronal T2-FLAIR, and perfusion weighted imaging during contrast agent injection.

### 2.2. Diffusion MRI

Diffusion MRI was performed with SE EPI sequence with the following scan parameters: TR/TE 6,683 ms/77 ms; 60 slices with a thickness of 2 mm; SENSE = 2; 128 × 128 matrix; FOV 256 × 256 mm; and diffusion encoding in 48 directions, with *b* = 1,000 s/mm^2^, for a total scan time of 6 minutes. All postprocessing was performed using in-house developed software, implemented in MATLAB (The Mathworks, Natick, MA, USA). Motion and eddy current distortions were corrected by registering the diffusion-weighted volumes to the volume acquired with *b* = 0 s/mm^2^, using ElastiX [41, 42]. In this process, the diffusion-weighted images were smoothed using 3D Gaussian kernel with a full width at half maximum of 2 mm. We used in-house developed software based on linear least-squares fitting with heteroscedasticity correction. Streamline tractography was performed using the Diffusion Toolkit and TrackVis (Ruopeng Wang, Van J. Wedeen, TrackVis.org, Martinos Center for Biomedical Imaging, Massachusetts General Hospital, MA, USA). The whole brain was seeded from two randomly positioned seeds in each voxel using FA threshold of 0.1 and an angular threshold of 45 degrees.

### 2.3. Tract Segmentation and Evaluation

**Figure 1:**
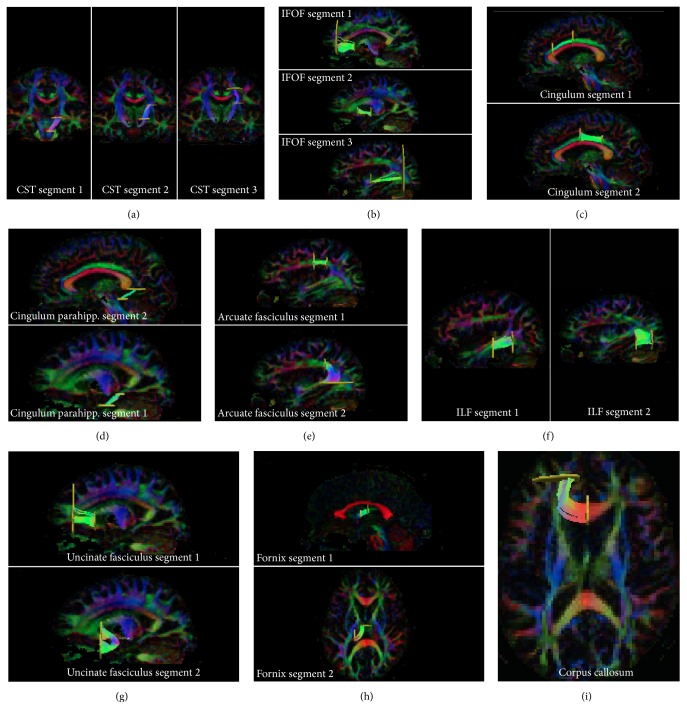
3D-tractography in TrackVis in all tract segments in one patient, in the hemisphere contralateral to the tumor. (a) Corticospinal tract: segments 1–3. (b) Inferior frontooccipital fasciculus: segments 1–3. (c) Cingulum: segments 1 and 2. (d) Parahippocampal cingulum: segments 1 and 2. (e) Arcuate fasciculus: segments 1 and 2. (f) Inferior longitudinal fasciculus: segments 1 and 2. (g) Uncinate fasciculus: segments 1 and 2. (h) Fornix: segments 1 and 2. (i) Corpus callosum: forceps minor.

ROIs (regions of interest) for tracking were delineated in EvalGUI, in-house developed software, based on earlier white matter atlas descriptions [43–45] and previous tractography evaluation of the normal brain [35]. Tracts were subdivided into segments that were defined based on anatomical landmarks, and ROI-delineation is visualized in Supplementary Figures 1 a-i (see Supplementary Material available online at http://dx.doi.org/10.1155/2016/7671854). These ROIs defined the anatomical landmarks. The ROI positions perpendicular to the tracts defined the segments. ROIs for each tract segment were positioned in the ipsilateral hemisphere and in the contralateral hemisphere in all patients; ROIs were adjusted when tract segment trajectory was altered due to tumor growth. TrackVis was used for visual confirmation of the tract positions (Figures 1(a)–1(i)).

**Figure 2:**
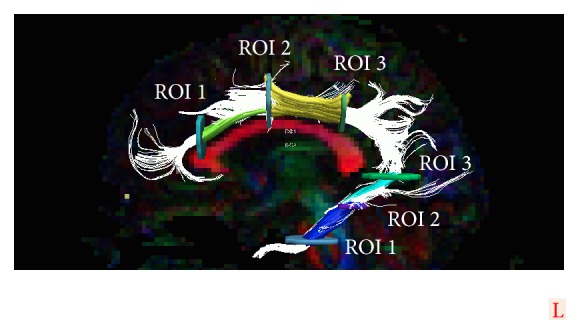
Example tract of cingulum with ROIs and tract segments. The cingulum and the parahippocampal part of the cingulum visualized as a 3D-tract with associated ROIs and tract segments overlaid on 2D FA-color map. Whole tract visualized in white. Cingulum: segment 1, green, and segment 2, yellow. Parahippocampal cingulum: segment 1, blue, and segment 2, turquoise.

Whole tracts were visually assessed in EvalGUI after selecting ROI positions. Tract segments were visually inspected in 3D using TrackVis.  Figure 2 shows an example of the cingulum with associated ROIs and tract segments.

The following tracts were evaluated in all patients: the corticospinal tract, the inferior frontooccipital fasciculus, the retrosplenial and parahippocampal parts of the cingulum [46], the arcuate fasciculus, the inferior longitudinal fasciculus, the uncinate fasciculus, fornix, and the forceps minor of the corpus callosum (Figures 1(a)–1(i)). Segments were numbered 1-2 or 1–3 from caudal to cranial ones, from anterior to posterior ones, or from midline to lateral ones depending on tract course (Figures 1(a)–1(i)). All tract segments were assessed for infiltration or dislocation by the tumor with reference standard for tumor area set as high signal intensity on T2-weighted images by a resident in neuroradiology with 5 years of experience in brain tumor evaluation. A tract segment was visually classified as infiltrated if it coursed through the tumor area defined as high signal intensity on T2-FLAIR weighted images. A tract segment was visually classified as dislocated if it deviated from its expected pathway on FA-color maps and was located outside the increased signal intensity on T2-FLAIR defined as tumor. Tumor location in the left or right hemisphere and lobes was determined on T2-weighted images.

### 2.4. Neuropathological Evaluation

All histopathological slides were reevaluated by one neuropathologist with extensive clinical experience of glioma diagnosis. The diagnoses followed the 2007 WHO classification of central nervous system tumors. The tumors were classified as either astrocytomas or oligodendrogliomas based on the dominating cell type [2].

### 2.5. Statistical Analysis

To standardize diffusion measurements, adjust for differences between hemispheres, and decrease interindividual variations between patients, a relative FA (rFA) was calculated for each tract segment by dividing the mean FA in the ipsilateral tract segment by the mean FA in the corresponding contralateral tract segment in each patient. The corresponding calculation was performed for mean MD, yielding relative MD (rMD). The FA and MD in tract segments are the mean values of all voxels in that segment. rFA and rMD were compared between groups based on radiographic features (tumor infiltration, tumor dislocation, and hemisphere location left/right) and neuropathological features (tumor type, tumor grade) with the Mann-Whitney *U* test. The Wilcoxon matched pairs test was used to evaluate differences in rFA between different segments of a tract partially infiltrated by tumor. The chi-squared test was used to investigate the distributions of visually infiltrating gliomas between grades II and III. The cut-off for significant results was set to a *p* value of <0.05. Statistical analyses were performed in Statistica version 12 (StatSoft, Dell Software, TX, USA).

## 3. Results

Thirty-four patients had tumors appearing low-grade on morphological MRI without (*n* = 24 patients) or with (*n* = 10 patients) patchy and faint contrast enhancement. Patient age at study entry was 48 ± 15 years (mean ± SD). The time between MRI and surgery was 3 ± 7 months (mean ± SD). The study population included 18 males and 16 females. Included patients had undergone presurgical radiological imaging, including morphological and physiological MRI with DTI (except for one patient, patient number 3, who had a biopsy 6 months prior to study MRI). Patients eligible for tractographic evaluation included in the statistical analysis were those with an astrocytoma (*n* = 18) or oligodendroglioma (*n* = 16) grade II or III according to postoperative neuropathological reevaluation. Patients with diagnosis other than astrocytoma or oligodendroglioma grade II or III on neuropathological assessment or patients not yet operated on were not included in the analysis. Tractography was successfully performed in 98% of the tract segments (635 tract segments out of 646, with 34 patients × 19 tract segments = 646). Technical details on tract segments regarding number of segments for each tract, segment length, and number of voxels per segment are presented in Supplementary Table 1. All segments except one were longer than 2 cm. A total of 19 segments from nine tracts were evaluated with DTT in each patient (Figures 1(a)–1(i)). The total number of segments in all tracts in each hemisphere was 19, and, out of these, 18 could be analyzed with rFA and rMD. rFA and rMD for segment one of the fornix could not be calculated because of its midline position.

### 3.1. Infiltration and Dislocation Based on Visual Assessment

On visual evaluation, 28 of the 34 included patients had at least one infiltrated tract segment (82%). Seventeen of the 34 patients (50%) had at least one dislocated tract segment. Thirteen of these 17 patients (76%) had other tract segments that were infiltrated on visual evaluation. Details concerning tract segment infiltration and dislocation and tumor location are presented in Table 1.

**Figure 3:**
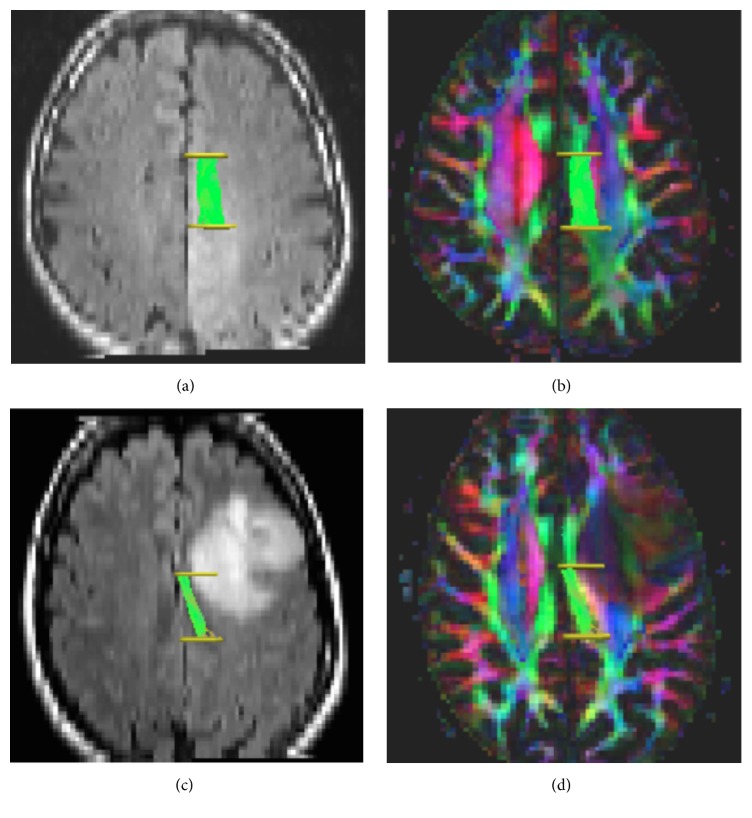
Tractography of segment 2 of the cingulum in two different patients (patient number 14 and patient number 25). ((a)-(b)) Patient with an astrocytoma grade III. (a) 3D-tractography of segment 2 of the cingulum overlaid on T2-FLAIR image shows tumor infiltrating the area of the tract segment. ROIs number two and three in yellow. (b) 3D-tractography in the same patient, segment two of the cingulum overlaid on 2D FA-color map. ((c)-(d)) Patient with an oligodendroglioma grade II. (c) 3D-tractography of segment 2 of the cingulum overlaid on T2-FLAIR image shows tumor dislocation of the tract segment. ROIs number two and three in yellow. (d) 3D-tractography in the same patient, segment two of the cingulum overlaid on 2D FA-color map.

Based on visual and quantitative evaluation, gliomas grade II and gliomas grade III had an equal propensity to infiltrate tract segments (chi-squared test, *p* = 0.75) and showed similar diffusion alterations (Supplementary Table 2). Figures 3(a)–3(d) show an example of one infiltrated and one dislocated tract in two different patients.

### 3.2. Relative FA and MD Compared with Visual Assessment and Neuropathological Report (Glioma Type Astrocytoma or Oligodendroglioma, Glioma Grade II or III)

rFA and rMD were calculated for all tract segments and compared between tract segments visually classified as infiltrated and those visually classified as not infiltrated. rFA was decreased in all tracts visually classified as infiltrated compared to those not classified as infiltrated, reaching statistical significance in all but two tract segments (89%, 16/18 segments), Table 2.

Similarly, rMD was higher in 89% of tract segments visually classified as infiltrated, reaching statistical significance in 78% (14/18 tract segments) (Table 3).

**Figure 4:**
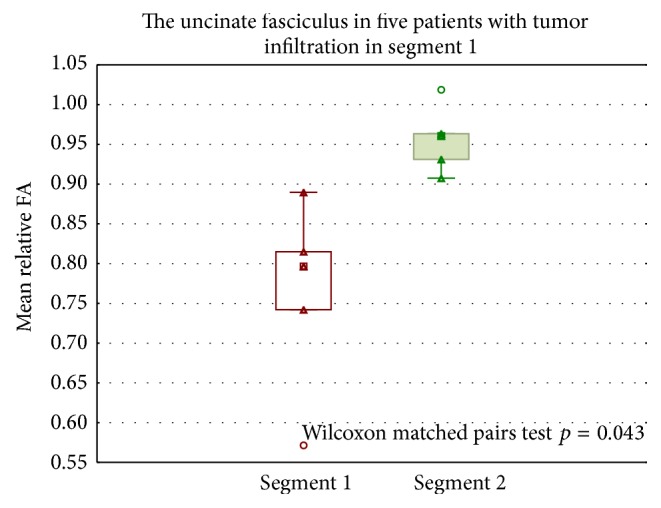
Box plot of rFA in tract segments. rFA in segments 1 and 2 in the uncinate fasciculus, in five patients with tumor infiltration in segment 1. Significantly lower rFA in segment 1 compared to segment 2. Wilcoxon matched pairs test, *p* = 0.043. rFA = relative fractional anisotropy.

Figure 4 shows a box plot of rFA and rMD in tract segment infiltration.

rFA was higher in one dislocated tract segment compared to the contralateral not dislocated tract segment, 6% (1/18). Tumor involvement often included several lobes (Table 1). In the involved lobes, several tract segments were often dislocated or infiltrated (Table 1). Furthermore, rFA and rMD were compared with neuropathological (glioma types astrocytoma and oligodendroglioma, glioma grades II and III) and radiological (visual tract segment dislocation or not, hemisphere location in right or left hemisphere) features. Detailed results from all comparisons are presented in Supplementary Table 2. rFA and rMD in tract segments did not differ significantly between gliomas grade II and gliomas grade III. Significant differences were found between rFA and rMD between astrocytomas (lower FA and higher MD) and oligodendrogliomas in 22% of all evaluated tract segments (4/18).

## 4. Discussion

Preoperative DTT evaluation of suspected tumor infiltration is increasingly required by the neurosurgeons, but it has not yet become a clinical routine procedure in every neuroradiological center. The method has been validated in normal populations but needs further evaluation in patients with suspected glioma to prove its clinical value [35]. We systematically assessed major associative, projection, and commissural bundles and compared quantitative analysis of tract segments with visual tract evaluation and neuropathological diagnosis in patients with nonnecrotic gliomas grades II and III. We divided the tracts into segments in order to improve the detection of partial tract infiltration.

Previous studies have reported on the usefulness of tractography for preoperative assessment of glioma infiltration along tracts with regard to surgical planning [11, 31]. Retrospective evaluation of gliomas has shown longer survival in patients with noninfiltrative gliomas [21, 47]. No prospective study has to our knowledge evaluated DTT in a cohort of prospectively included suspected low-grade gliomas. Our main finding was that radiological suspicion of glioma infiltration on visual evaluation was confirmed by significantly reduced rFA in 89% (16/18) of the evaluated tract segments. 11% (2/18) had lower rFA but did not reach statistical significance. In segment two of the fornix only two out of 32 patients had visual infiltration making the comparison less likely to show statistical significance. In consistency with earlier reports, dislocated tract segments had no significant alterations of rFA or rMD in 94% compared with not dislocated tract segments, Supplementary Table 2.

Reduced FA in tumor-infiltrated white matter regions is the most studied finding related to DTI tumor evaluation. Supported by DTI experiment in rat glioma models, reduced rFA in investigated tract segments indicates glioma growth [19]. In general, rFA and rMD in tract segments showed a poor association with glioma grade and type in our study. Gliomas grades II and III had an equal distribution of visual tract segment infiltration, highlighting the intrinsic propensity for infiltrative growth in both tumor grades.

In consistency with earlier reports on glioma infiltration and dislocation of tracts, several patients had infiltration of some tracts combined with dislocation of others [47]. Why gliomas infiltrate some tracts and dislocate others has still to be elucidated. The vulnerability of specific white matter tracts, tumor location, neuropathological glioma characteristics, and progressive disease or tumor volume could all play a role in the processes that influence gliomas growth.

Suspected low-grade gliomas share common imaging criteria: no or patchy and faint contrast enhancement, no extensive mass effect, and no edema. These inclusion criteria, as well as earlier studies showing that low-grade gliomas are mainly confined within the hyperintensity on T2-weighted images, support that our findings of lower rFA and higher rMD in infiltrated segments are related to tumor growth, and not edema. The fact that these tumors (suspected low-grade) do not exhibit surrounding edema makes the changes in diffusion found in visually infiltrated tracts most likely due to bulk tumor infiltration.

The strengths of this study are attributed to the extensive analysis of 19 segments in nine white matter pathways in a prospectively gathered cohort of patients with suspected low-grade gliomas. Segment-wise tractography rather than averaging across a whole tract increases the regional specificity. All patients included in the analysis had a confirmed neuropathological diagnosis of astrocytoma or oligodendroglioma grade II or grade III.

Preoperative knowledge about suspected tumor tract infiltration and tumor tract dislocation can aid surgical preoperative planning and indicates the need for an extended preoperative evaluation including also neurophysiological assessment. It might also aid upon informing the patient about the expected neurological outcome after surgery, which may depend on the extent of tract involvement.

Based on our findings, future studies evaluating measurement of diffusion properties in multiple tract segments may provide new important information about the extent of glioma infiltration. Future applications of this method might also give insight into tract segment infiltration related to different histological subtypes of gliomas.

We acknowledge three major limitations of the present study. First, the limited number of patients included in this study is a disadvantage in a study of heterogeneous tumors with different locations in the brain. Small differences in subgroups can be missed, and our findings need to be interpreted with care and need confirmation in larger cohorts [32]. However, the results on diffusion changes in visually infiltrated tract segments were a consistent finding over different patients and different tracts segments.

Second, infiltrated tracts were visually classified based on T2-FLAIR and FA-color maps; however, this study lacks neuropathological verifications of these findings. To evaluate the visual assessment of tract segment infiltration we compared the results from visual analysis with quantitative data from tract segments. In support of our findings regarding low rFA in infiltrated tract segments, Stadlbauer et al. 2007 showed an association between lower FA values and increasing tumor cell numbers [48].

The third limitation concerns the tractography method. A weakness of the DTT technique is the difficulty of successful tractography in regions with crossing fibers [49, 50]. We strove to minimize this influence by avoiding tracts and segments with known difficulties when deterministic tractography is used. In the segmentation of the tracts, we balanced segment length and tractography quality, and, thus, a majority of investigated tracts (7/9) comprised two segments. This limited the possibility of studying variation in FA and MD along tracts and at different distances from the tumor. Also, FA is affected by both white matter integrity and orientation dispersion and methods to separate the two have been devised recently but require high *b*-value data [51].

Since intraoperative subcortical mapping using direct electrical stimulations under awake surgery is the gold standard to directly visualize the subcortical connectivity future DTT studies should be compared with intraoperative observation of subcortical tracts [9].

## 5. Conclusion

Quantitative MR tractography is applicable in patients with nonnecrotic gliomas grades II and III and corresponds to visual evaluation of suspected tract infiltration. It is thus a promising objective tool for the preoperative evaluation of tract segment involvement in these tumors.

## Supplementary Material

Supplementary figures 1 a-i: ROI delineation and position.
Supplementary table 1: Tractography details.
Supplementary table 2: Full tractography results.

## Figures and Tables

**Table 1:** A summary of infiltrated or dislocated tract segments.

Patient number	Glioma type	Glioma grade	Lobe	Displaced tracts	Displaced segments	Infiltrated tracts	Infiltrated segments
1	Astrocytoma	II	Parietal	None	None	AF	AF segments 1 and 2

2	Astrocytoma	III	Frontal lobe, not extending to the motor area	None	None	None	None

3	Astrocytoma	II	Frontoparietotemporal	None	None	ILF IFOF CST CG CG para AF Fornix	ILF segment 2 IFOF segment 3 CST segments 2 and 3 CG segments 1 and 2 CG para segment 2 AF segments 1 and 2 Fornix segment 2

4	Oligodendroglioma	II	Frontal lobe, not extending to the motor area	None	None	UF IFOF CC Fmin	UF segment 1 IFOF segment 1 CC Fmin

5	Astrocytoma	II	Frontal and insular	IFOF AF CST	IFOF segment 2 AF segment 1 CST segments 2 and 3	None	None

6	Astrocytoma	III	Frontal lobe extending to the precentral sulcus	CST	CST segments 2 and 3	AF	AF segments 1 and 2

7	Astrocytoma	III	Temporooccipital	CST	CST segment 2	UF ILF IFOF CG para Fornix	UF segments 1 and 2 ILF segments 1 and 2 IFOF segments 2 and 3 CG para segments 1 and 2 Fornix segment 2

8	Astrocytoma	III	Frontoparietotemporal	ILF IFOF CST	ILF segment 2 IFOF segment 3 CST segments 2 and 3	AF	AF segments 1 and 2

9	Oligodendroglioma	II	Frontal lobe, not extending to the motor area	None	None	UF IFOF CC Fmin CG	UF segments 1 and 2 IFOF segments 1 and 2 CC Fmin CG segments 1 and 2

10	Oligodendroglioma	II	Frontal	CG	CG segments 1 and 2	CST AF	CST segments 2 and 3 AF segment 1

11	Oligodendroglioma	II	Parietal	None	None	None	None

12	Astrocytoma	III	Frontal lobe, not extending to the motor area	None	None	UF IFOF CC Fmin	UF segments 1 and 2 IFOF segment 1 CC Fmin

13	Astrocytoma	II	Frontoparietal	AF	AF segment 1	CST CG	CST segment 3 CG segments 1 and 2

14	Astrocytoma	III	Frontotemporoparietooccipital	ILF IFOF	ILF segments 1 and 2 IFOF segment 3	CST CG CG para AF	CST segment 3 CG segments 2 and 1 CG para segments 1 and 2 AF segment 2

15	Astrocytoma	III	Frontoparietotemporal	None	None	AF	AF segments 1 and 2

16	Astrocytoma	II	Parietotemporooccipital	None	None	IFOF CST AF	IFOF segment 3 CST segment 3 AF segment 2

17	Oligodendroglioma	II	Temporal	CG para	CG para segment 1	UF IFOF	UF segments 1 and 2 IFOF segment 2

18	Oligodendroglioma	II	Frontal lobe, not extending to the motor area	None	None	CC Fmin	CC Fmin

19	Astrocytoma	II	Frontotemporoparietal	None	None	UF ILF IFOF CG para	UF segments 1 and 2 ILF segments 1 and 2 IFOF segments 2 and 3 CG para segments 1 and 2

20	Oligodendroglioma	II	Frontal	AF	AF segment 1	None	None

21	Astrocytoma	II	Frontal	CG	CG segments 1 and 2	None	None

22	Oligodendroglioma	II	Temporoparietal	ILF IFOF	ILF segment 1 IFOF segment 3	None	None

23	Astrocytoma	II	Frontotemporoinsular	CST	CST segments 1 and 2	UF ILF IFOF	UF segments 1 and 2 ILF segment 1 IFOF segments 2 and 3

24	Astrocytoma	II	Temporooccipitoparietal	None	None	ILF IFOF CST ARC	ILF segments 1 and 2 IFOF segment 3 CST segments 2 and 3 FA segments 1 and 2

25	Oligodendroglioma	II	Frontal lobe extending to the precentral sulcus	CG	CG segments 1 and 2	CST ARC	CST segments 2 and 3 AF segment 1

26	Astrocytoma	III	Frontotemporoparietooccipital	CST	CST segments 1 and 2	UF ILF IFOF CG para ARC	UF segments 1 and 2 ILF segments 1 and 2 IFOF segments 1–3 CG para segments 1 and 2 AF segment 2

27	Oligodendroglioma	II	Frontal lobe, not extending to the motor area	CG	CG segment 1	UF CC Fmin	UF segments 1 and 2

28	Oligodendroglioma	II	Frontal lobe extending to the precentral sulcus	CST	CST segments 2 and 3	AF	AF segment 1

29	Oligodendroglioma	II	Frontotemporoinsular	AF CST	AF segments 1 and 2 CST segments 2 and 3	UF ILF IFOF	UF segments 1 and 2 ILF segment 1 IFOF segments 1–3

30	Oligodendroglioma	III	Frontal	None	None	CST	CST segment 3

31	Oligodendroglioma	II	Frontal lobe, not extending to the motor area	None	None	CC Fmin	CC Fmin

32	Oligodendroglioma	III	Frontotemporoinsular	None	None	UF ILF IFOF CC Fmin	UF segments 1 and 2 ILF segment 1 IFOF segments 1–3 CC Fmin

33	Astrocytoma	III	Frontoinsular, not extending to the precentral sulcus	None	None	UF IFOF CC Fmin	UF segment 1 IFOF segments 1 and 2 CC Fmin

34	Oligodendroglioma	III	Frontoinsular, not extending to the precentral sulcus	None	None	UF IFOF CC Fmin	UF segment 1 IFOF segments 1 and 2 CC Fmin

AF = arcuate fasciculus, ILF = inferior longitudinal fasciculus, IFOF = inferior frontooccipital fasciculus, CG = cingulum, CG para = parahippocampal cingulum, UF = uncinate fasciculus, CC Fmin = forceps minor of the corpus callosum, and CST = corticospinal tract.

**Table 2:** Results from tractography, fractional anisotropy.

Track		Corticospinal tract	*p*		Corticospinal tract	*p*		Corticospinal tract	*p*

DTI scalars		rFA mean (SD)			rFA mean (SD)			rFA mean (SD)	
Segment		Segment 1			Segment 2			Segment 3	
Infiltrated tract segment	*n* = 8	0.96 (0.03)	**0.006135**	*n* = 8	0.93 (0.06)	**0.021797**	*n* = 8	0.95 (0.08)	0.138
Not infiltrated tract segment	*n* = 26	1.01 (0.05)		*n* = 26	1.00 (0.06)		*n* = 26	1.01 (0.08)	

Track		Inferior frontooccipital fasciculus	*p*		Inferior frontooccipital fasciculus	*p*		Inferior frontooccipital fasciculus	*p*

DTI scalars		rFA mean (SD)			rFA mean (SD)			rFA mean (SD)	
Segment		Segment 1			Segment 2			Segment 3	
Infiltrated tract segment	*n* = 14	0.80 (0.14)	**0.00029**	*n* = 15	0.74 (0.16)	**0.000027**	*n* = 14	0.89 (0.11)	**0.000218**
Not infiltrated tract segment	*n* = 19	0.99 (0.07)		*n* = 19	0.98 (0.08)		*n* = 19	1.03 (0.06)	

Track		Cingulum	*p*		Cingulum	*p*		

DTI scalars		rFA mean (SD)			rFA mean (SD)				
Segment		Segment 1			Segment 2				
Infiltrated tract segment	*n* = 4	0.64 (0.16)	**0.004241**	*n* = 4	0.58 (0.22)	**0.001471**			
Not infiltrated tract segment	*n* = 30	1.02 (0.17)		*n* = 30	1.03 (0.07)				

Track		Parahippocampal cg	*p*		Parahippocampal cg	*p*		

DTI scalars		rFA mean (SD)			rFA mean (SD)				
Segment		Segment 1			Segment 2				
Infiltrated tract segment	*n* = 5	0.61 (0.21)	**0.002188**	*n* = 5	0.55 (0.14)	**0.000463**			
Not infiltrated tract segment	*n* = 29	0.98 (0.10)		*n* = 29	0.98 (0.11)				

Track		Arcuate fasciculus	*p*		Arcuate fasciculus	*p*			

DTI scalars		rFA mean (SD)			rFA mean (SD)				
Segment		Segment 1			Segment 2				
Infiltrated tract segment	*n* = 12	0.76 (0.16)	**0.000386**	*n* = 10	0.76 (0.11)	**0.000026**			
Not infiltrated tract segment	*n* = 22	0.98 (0.06)		*n* = 21	1.00 (0.07)				

Track		Inferior longitudinal fasciculus	*p*		Inferior longitudinal fasciculus	*p*		

DTI scalars		rFA mean (SD)			rFA mean (SD)				
Segment		Segment 1			Segment 2				
Infiltrated tract segment	*n* = 8	0.75 (0.18)	**0.000173**	*n* = 8	0.84 (0.09)	**0.000173**			
Not infiltrated tract segment	*n* = 26	0.99 (0.08)		*n* = 26	1.00 (0.07)				

Track		Uncinate fasciculus	*p*		Uncinate fasciculus	*p*		

DTI scalars		rFA mean (SD)			rFA mean (SD)				
Segment		Segment 1			Segment 2				
Infiltrated tract segment	*n* = 12	0.76 (0.09)	**0.000003**	*n* = 12	0.77 (0.18)	**0.001058**			
Not infiltrated tract segment	*n* = 21	1.01 (0.08)		*n* = 21	1.00 (0.10)				

Track		Fornix	*p*		Fornix	*p*		

DTI scalars		FA ratio			rFA mean (SD)				
Segment		1, midline			Segment 2				
Infiltrated tract segment	NA^*∗*^	NA^*∗*^	NA^*∗*^	*n* = 2	0.88 (0.10)	0.056			
Not infiltrated tract segment	NA^*∗*^	NA^*∗*^	NA^*∗*^	*n* = 30	1.02 (0.09)				

Track		Callosal body minor forceps	*p*					

DTI scalars		rFA mean (SD)							
Segment		Segment 1							
Infiltrated tract segment	*n* = 9	0.88 (0.12)	**0.00301**						
Not infiltrated tract segment	*n* = 25	1.01 (0.05)							

^*∗*^No ratio due to tract segment in midline.

rFA in infiltrated and not infiltrated tract segment presented as mean and standard deviation with results from Mann-Whitney *U* tests. *p* < 0.05 was regarded as statistically significant.

rFA = relative fractional anisotropy (ipsilateral mean FA/contralateral mean FA), SD = standard deviation, cg = cingulum, and NA = not available.

**Table 3:** Results from tractography, mean diffusivity.

Track		Corticospinal tract	*p*		Corticospinal tract	*p*		Corticospinal tract	*p*

DTI scalars		rMD mean (SD)			rMD mean (SD)			rMD mean (SD)	
Segment		Segment 1			Segment 2			Segment 3	
Infiltrated tract segment	*n* = 8	0.98 (0.05)	0.383	*n* = 8	1.01 (0.04)	0.761	*n* = 8	1.08 (0.10)	**0.009935**
Not infiltrated tract segment	*n* = 26	0.99 (0.04)		*n* = 26	1.01 (0.04)		*n* = 26	1.00 (0.04)	

Track		Inferior frontooccipital fasciculus	*p*		Inferior frontooccipital fasciculus	*p*		Inferior frontooccipital fasciculus	*p*

DTI scalars		rMD mean (SD)			rMD mean (SD)			rMD mean (SD)	
Segment		Segment 1			Segment 2			Segment 3	
Infiltrated tract segment	*n* = 14	1.19 (0.21)	**0.000122**	*n* = 15	1.20 (0.26)	**0.009287**	*n* = 14	1.07 (0.09)	**0.018801**
Not infiltrated tract segment	*n* = 19	1.00 (0.02)		*n* = 19	1.01 (0.04)		*n* = 19	0.99 (0.06)	

Track		Cingulum	*p*		Cingulum	*p*		

DTI scalars		rMD mean (SD)			rMD mean (SD)				
Segment		Segment 1			Segment 2				
Infiltrated tract segment	*n* = 4	1.19 (0.15)	**0.002116**	*n* = 4	1.47 (0.55)	**0.001471**			
Not infiltrated tract segment	*n* = 30	1.03 (0.04)		*n* = 30	1.01 (0.04)				

Track		Parahippocampal cg	*p*		Parahippocampal cg	*p*		

DTI scalars		rMD mean (SD)			rMD mean (SD)				
Segment		Segment 1			Segment 2				
Infiltrated tract segment	*n* = 5	1.44 (0.34)	**0.001574**	*n* = 5	1.37 (0.25)	**0.000793**			
Not infiltrated tract segment	*n* = 29	1.03 (0.10)		*n* = 29	1.01 (0.08)				

Track		Arcuate fasciculus	*p*		Arcuate fasciculus	*p*			

DTI scalars		rMD mean (SD)			rMD mean (SD)				
Segment		Segment 1			Segment 2				
Infiltrated tract segment	*n* = 12	1.34 (0.28)	**0.000166**	*n* = 10	1.22 (0.21)	**0.001226**			
Not infiltrated tract segment	*n* = 22	1.00 (0.04)		*n* = 21	1.01 (0.04)				

Track		Inferior longitudinal fasciculus	*p*		Inferior longitudinal fasciculus	*p*		

DTI scalars		rMD mean (SD)			rMD mean (SD)				
Segment		Segment 1			Segment 2				
Infiltrated tract segment	*n* = 8	1.19 (0.15)	**0.000602**	*n* = 8	1.11 (0.11)	**0.002844**			
Not infiltrated tract segment	*n* = 26	1.01 (0.04)		*n* = 26	1.00 (0.06)				

Track		Uncinate fasciculus	*p*		Uncinate fasciculus	*p*		

DTI scalars		rMD mean (SD)			rMD mean (SD)				
Segment		Segment 1			Segment 2				
Infiltrated tract segment	*n* = 12	1.18 (0.15)	**0.000146**	*n* = 12	1.24 (0.27)	0.15			
Not infiltrated tract segment	*n* = 21	1.01 (0.03)		*n* = 21	1.01 (0.04)				

Track		Fornix	*p*		Fornix	*p*		

DTI scalars		rMD mean (SD)			rMD mean (SD)				
Segment		1, midline			Segment 2				
Infiltrated tract segment	NA^*∗*^	NA^*∗*^	NA^*∗*^	*n* = 2	1.09 (0.02)	0.371			
Not infiltrated tract segment	NA^*∗*^	NA^*∗*^	NA^*∗*^	*n* = 30	1.03 (0.19)				

Track		Callosal body minor forceps	*p*					

DTI scalars		rMD mean (SD)							
Segment		Segment 1							
Infiltrated tract segment	*n* = 9	1.13 (0.08)	**0.000209**						
Not infiltrated tract segment	*n* = 25	1.01 (0.02)							

^*∗*^No ratio due to tract segment in midline.

rMD in infiltrated and not infiltrated tract segment presented as mean and standard deviation with results from Mann-Whitney *U* tests. *p* < 0.05 was regarded as statistically significant.

rMD = relative mean diffusivity (ipsilateral mean diffusivity/contralateral mean diffusivity), SD = standard deviation, cg = cingulum, and NA = not available.

## Abbreviations

rFA: Relative fractional anisotropy
rMD: Relative mean diffusivity
DTT: Diffusion tensor tractography
DTI: Diffusion tensor imaging
ROI: Region of interest.
