# Supplementary material for: Preoperative Quantitative MR Tractography Compared with Visual Tract Evaluation in Patients with Neuropathologically Confirmed Gliomas Grades II and III: A Prospective Cohort Study
Source: Radiol Res Pract. 2016 Apr 17;2016:7671854. doi: 10.1155/2016/7671854 (PMC4852118; doi:10.1155/2016/7671854)
Supplement: Supplementary file 1 — Supplementary figures 1 a-i: ROI delineation and position. Supplementary table 1: Tractography details. Supplementary table 2: Full tractography results. [file 7671854.f1.pdf]

Supplementary information

S. fig 1 a-i

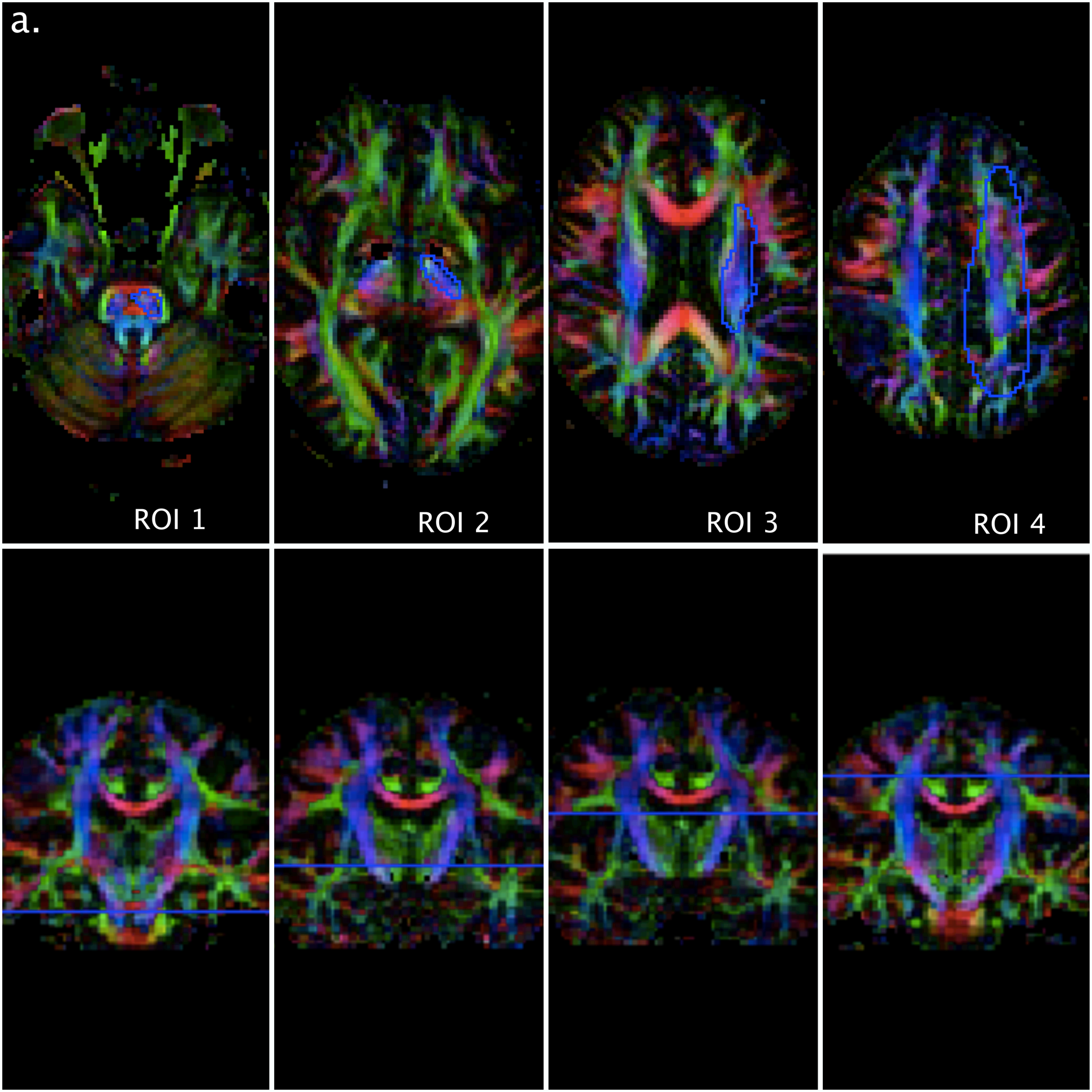

a.
ROI 1
ROI 2
ROI 3
ROI 4

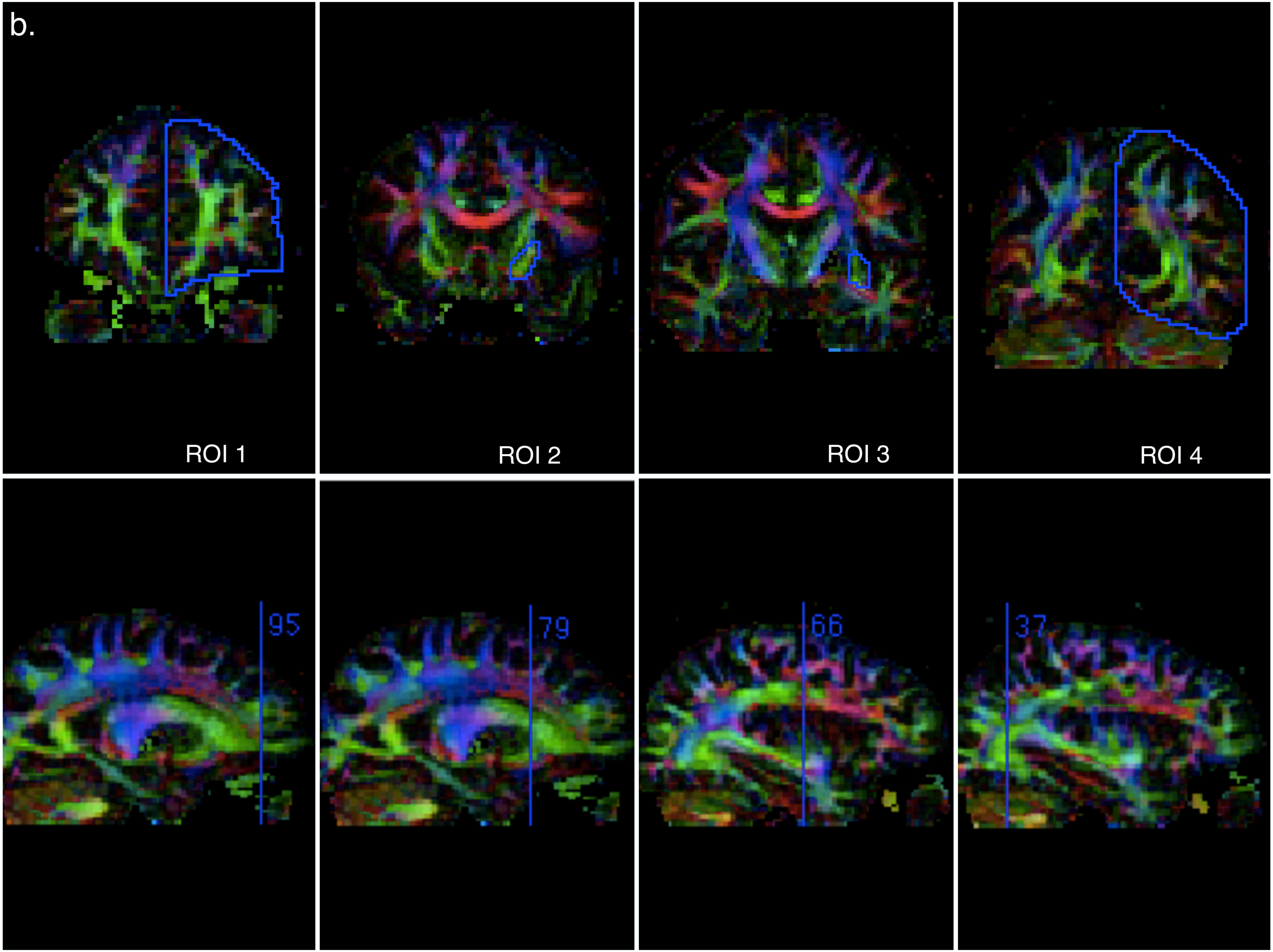
b.
ROI 1
ROI 2
ROI 3
ROI 4
95
79
66
37

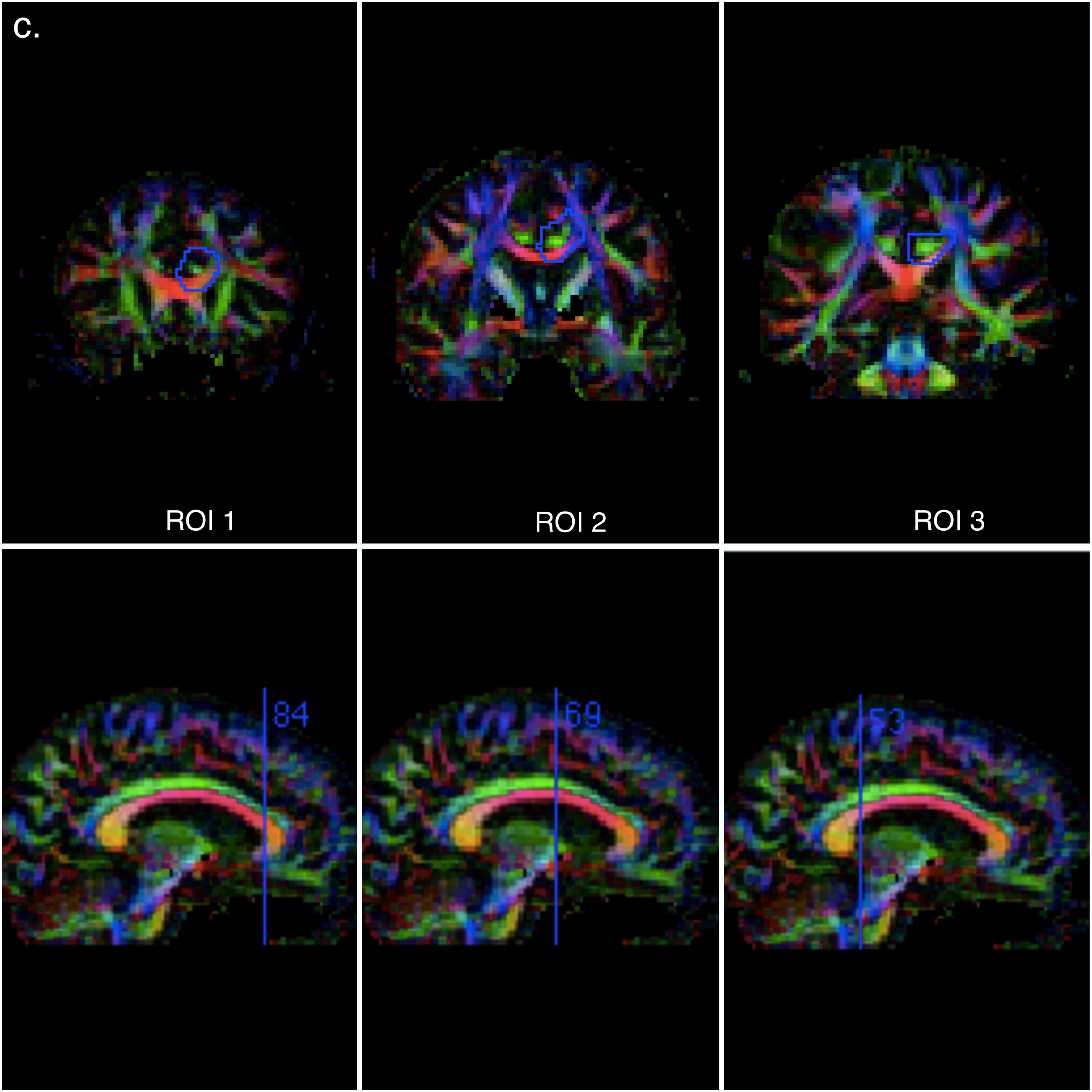

c.
ROI 1
ROI 2
ROI 3
84
69
53

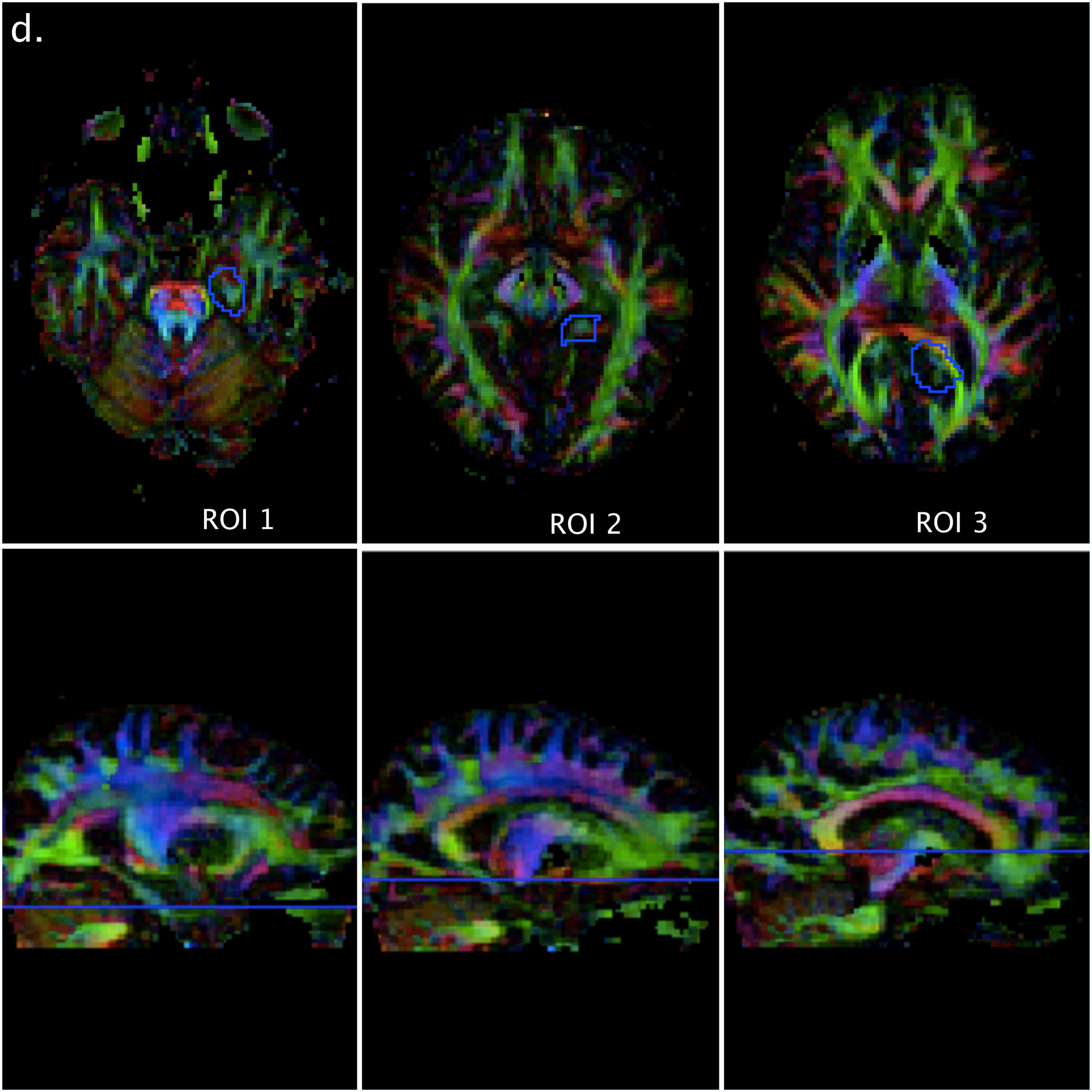
d.
ROI 1
ROI 2
ROI 3

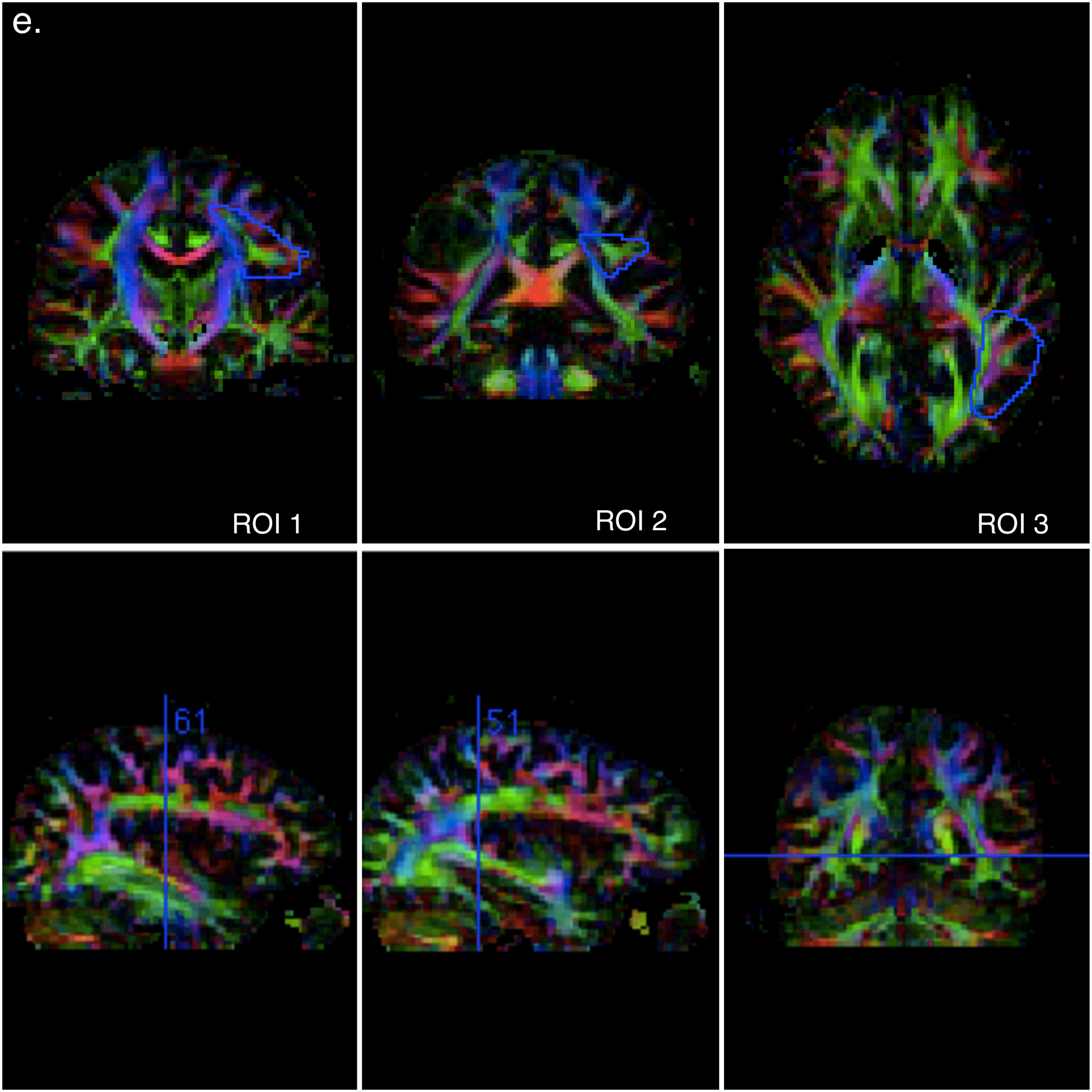
e.
ROI 1
ROI 2
ROI 3
61
51

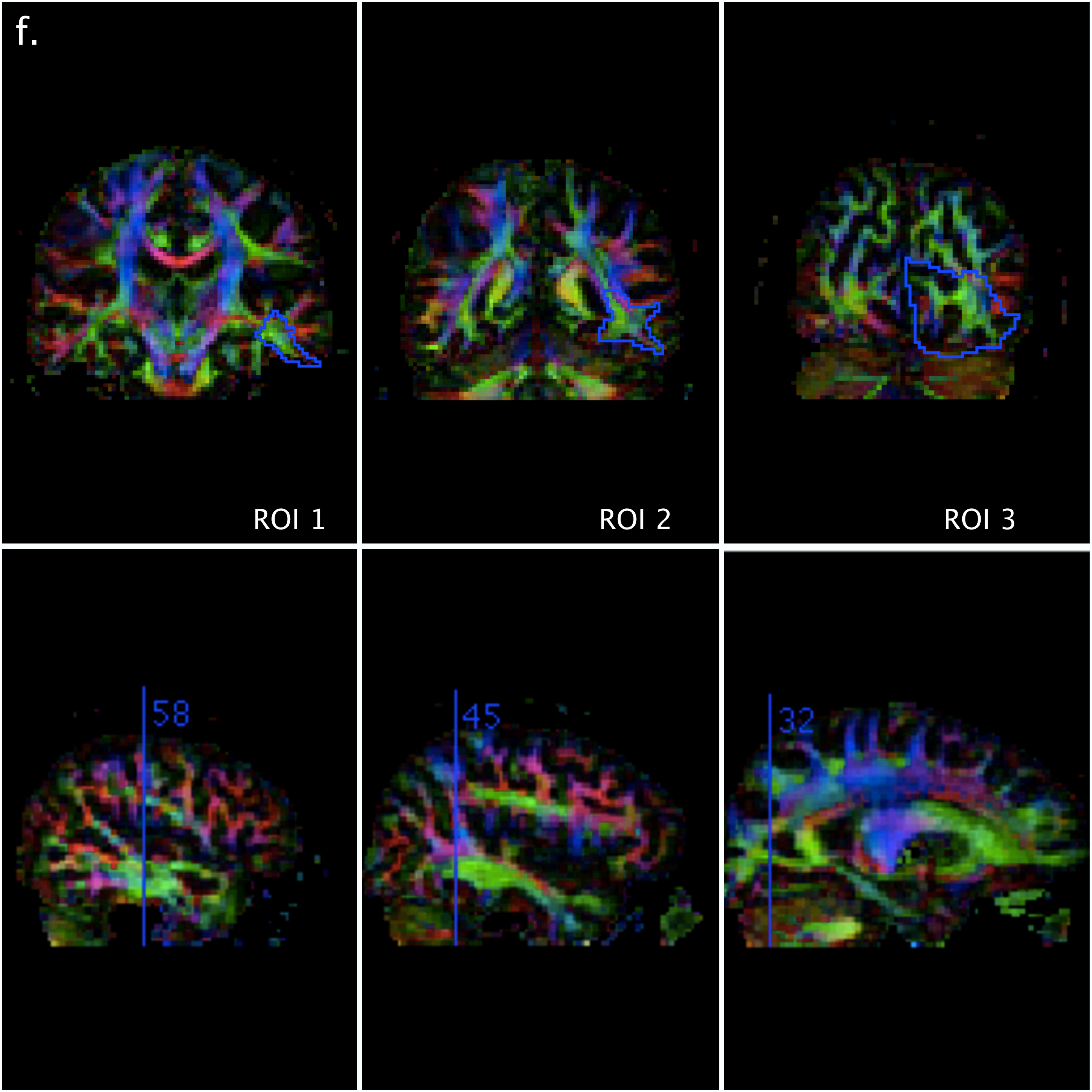
f.
ROI 1
ROI 2
ROI 3
58
45
32

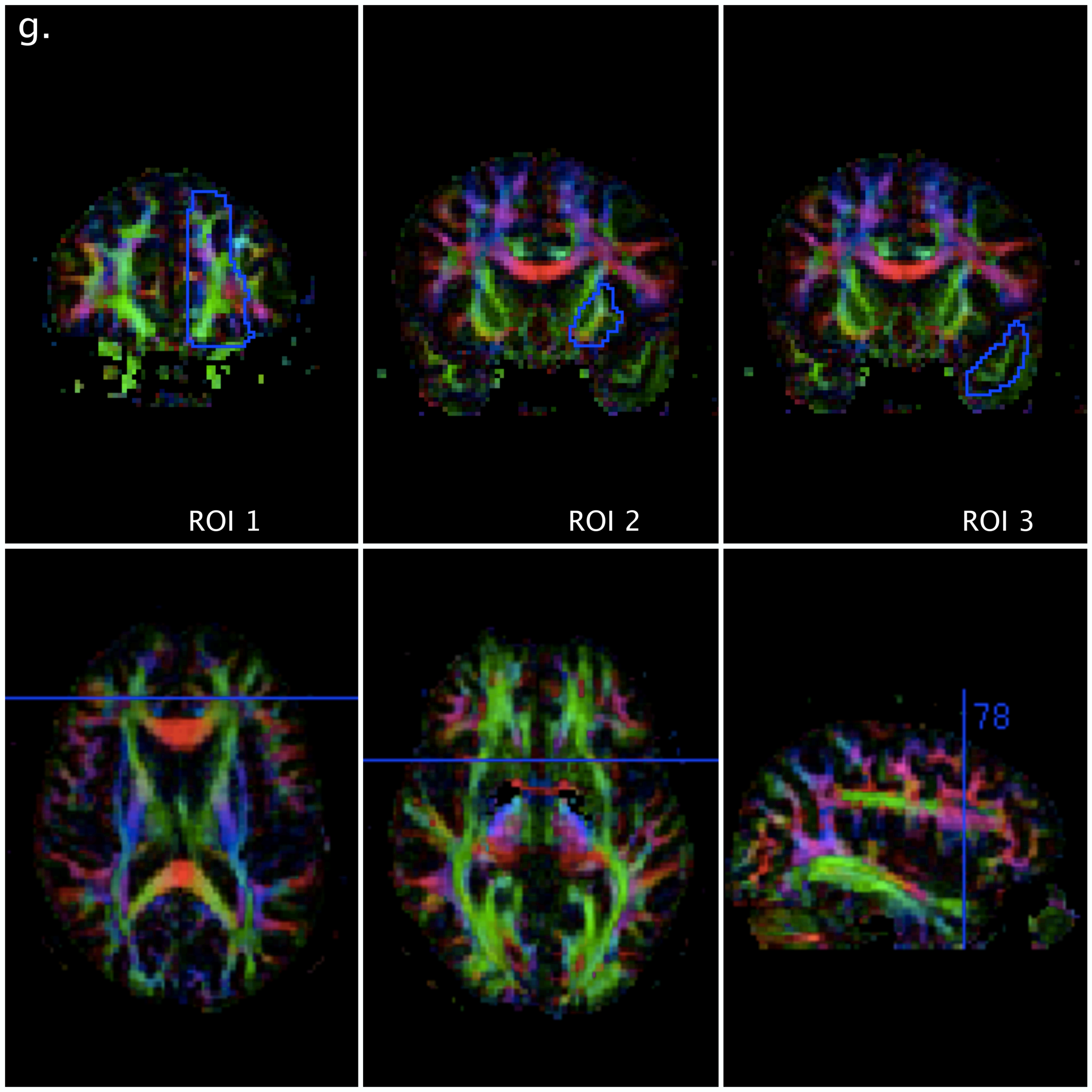
g.
ROI 1
ROI 2
ROI 3
78

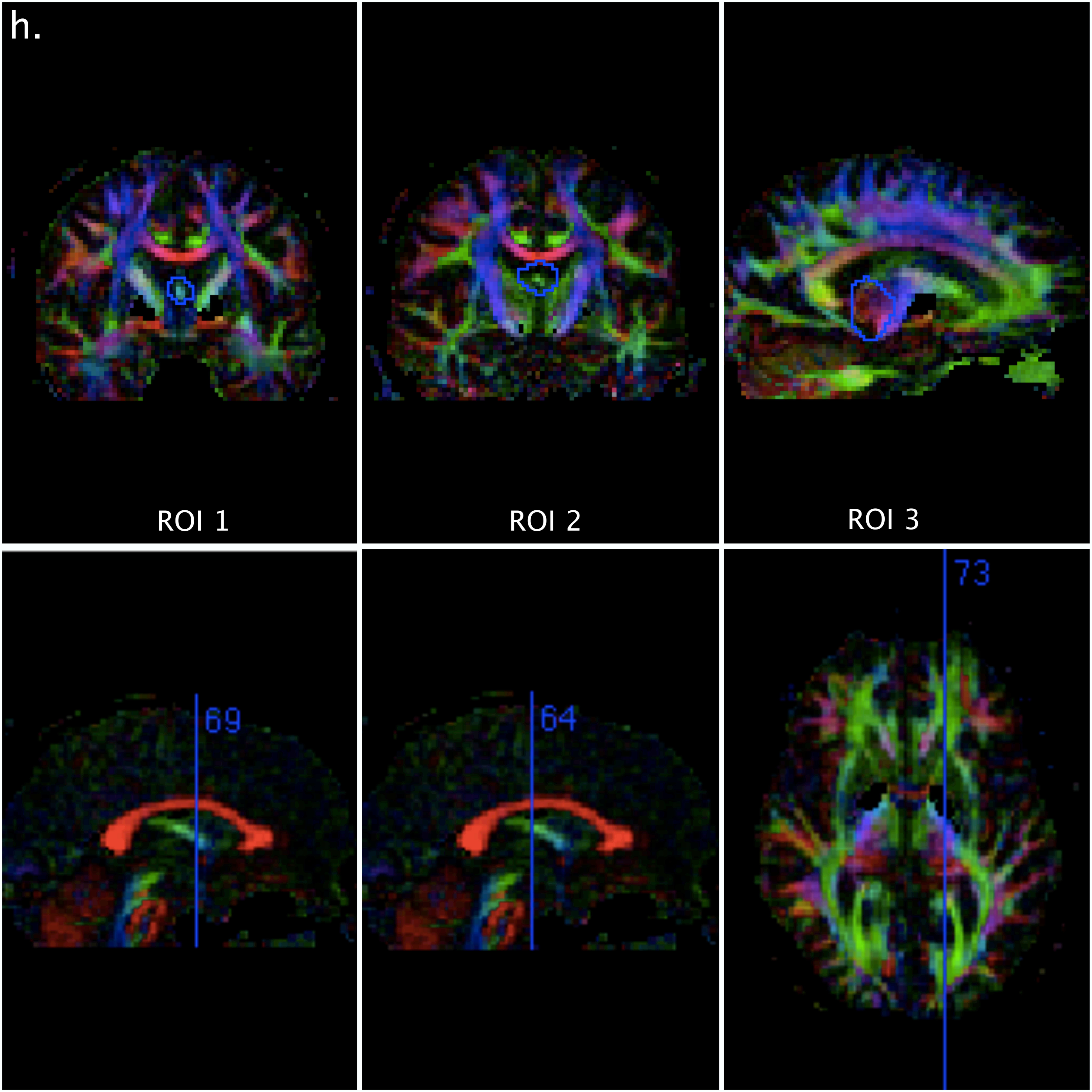
h.
ROI 1
ROI 2
ROI 3
69
64
73

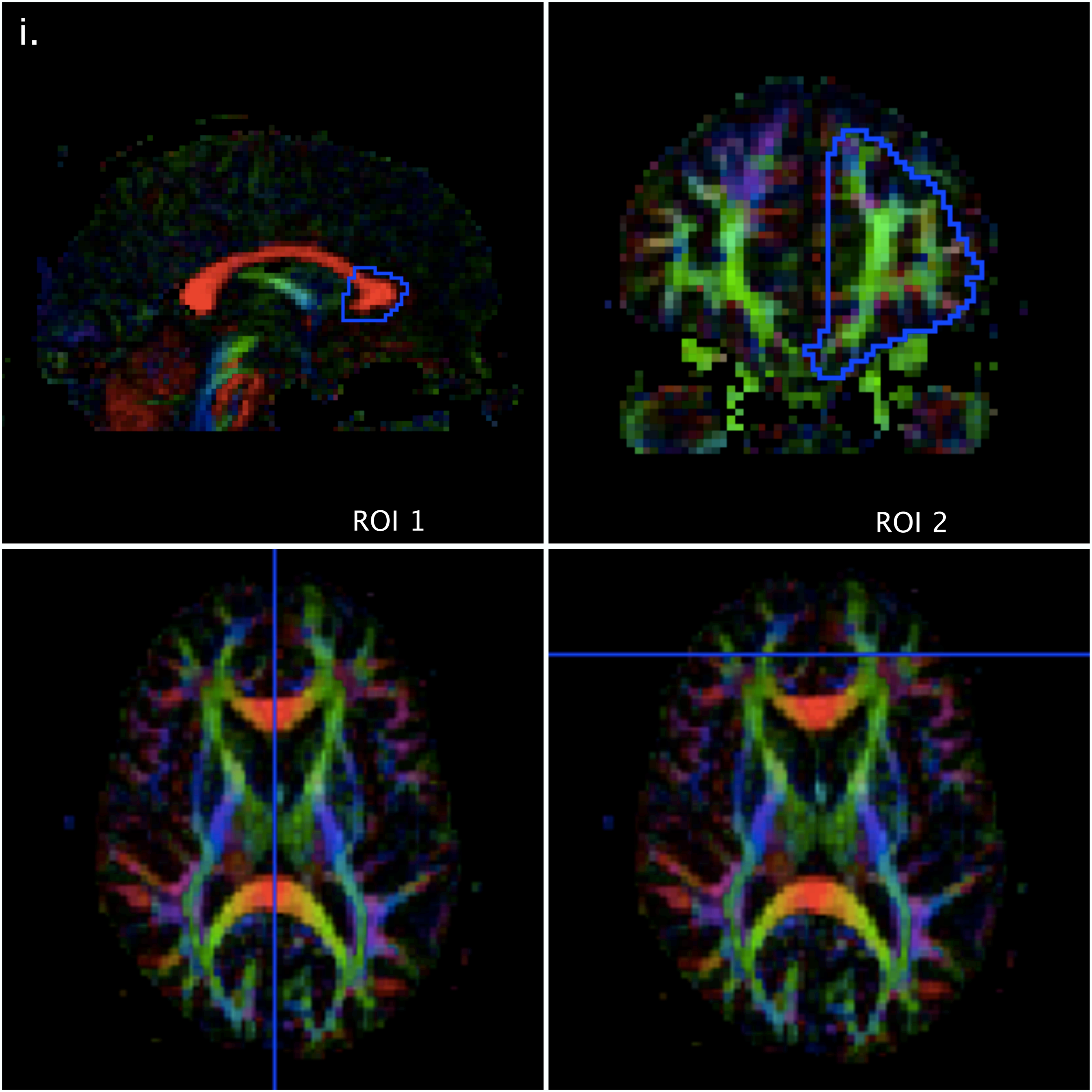
i.
ROI 1
ROI 2

Supplementary images 1 a-i. Regions of interest delineation (ROI) = anatomical landmarks for all tract segments in one hemisphere contralateral to the tumor, overlaid on FA-color maps.

Upper images contain delineated ROIs while lower images indicate the position on an orthogonal slice.

1a. Cortico-spinal tract. ROI 1: Superior level of the pons. ROI 2: Inferior part of the posterior limb of the internal capsule at the horizontal level of the anterior commissure. ROI 3: Superior part of the posterior limb of the internal capsule. ROI 4: Superior to the cingulum in the corona radiata.

1b. Inferior fronto-occipital fasciculus. ROI 1: Anterior to the genu of the callosal body. ROI 2: Anterior aspect of the external capsule. ROI 3: Posterior to the anterior commissure in the external capsule. ROI 4: Posterior to the splenium of the callosal body.

1c. Cingulum. ROI 1: Superior to the anterior part of the callosal body. ROI 2: Superior to the middle part of the callosal body. ROI 3: Superior to the posterior part of the callosal body.

1d. Parahippocampal part of the cingulum. ROI 1: Medially in the temporal lobe at the level of the superior part of the pons. ROI 2: Parasagittal, superior to the cerebellum. ROI 3: Inferior to the splenium of the callosal body.

1e. Arcuate fasciculus. ROI 1: Lateral to the corona radiata, at a vertical level of the thalamus. ROI 2: Posterior to ROI number one, at a vertical level of the anterior aspect of the cerebellum. ROI 3: In the occipito-temporal region at the level of the thalamus.

1f. Inferior longitudinal fasciculus. ROI 1: In the temporal lobe at the horizontal level of the mesencephalon. ROI 2: In the occipito-temporal region at the horizontal level of the thalamus.

ROI 3: In the occipital lobe at the horizontal level of the mesencephalon.

1g. Uncinate fasciculus. ROI 1: Anterior to genu of the callosal body. ROI 2: Anterior region of the external capsule. ROI 3: Anterior region of the temporal lobe. A negative ROI (not-ROI) was positioned posterior to ROI number two.

1h. Fornix. ROI 1: Midline, below the callosal body. ROI 2: posterior to ROI number one. ROI 3: Below the thalamus.

1i. Forceps minor of the callosal body. ROI 2: Genu of the callosal body. ROI 2: Anterior to the genu of the callosal body.

Supplementary table 1. Tractography detalis

Tract	Segment	Side	N voxels mean (SD)	Length mm mean (SD)
Corticospinal tract	Segment 1	Ipsilateral	342 (91)	27 (2)
		Contralateral	374 (89)	28 (1)
	Segment 2	Ipsilateral	712 (152)	26 (1)
		Contralateral	721 (199)	25 (1)
	Segment 3	Ipsilateral	735 (176)	26 (2)
		Contralateral	775 (164)	25 (1)
Inferior fronto-occipital fasciculus	Segment 1	Ipsilateral	356 (143)*	35 (2)*
		Contralateral	409 (124)*	35 (1)*
	Segment 2	Ipsilateral	236 (73)	30 (2)
		Contralateral	219 (48)	30 (1)
	Segment 3	Ipsilateral	541 (267)†	63 (4)†
		Contralateral	661 (214)†	63 (2)†
Cingulum	Segment 1	Ipsilateral	176 (40)	32 (3)
		Contralateral	183 (41)	32 (3)
	Segment 2	Ipsilateral	231 (54)	32 (3)
		Contralateral	235 (62)	32 (3)
Parahippocampal cingulum	Segment 1	Ipsilateral	151 (42)	22 (3)
		Contralateral	151 (34)	23 (2)
	Segment 2	Ipsilateral	102 (32)	25 (4)
		Contralateral	107 (25)	25 (2)
Arcuate fasciculus	Segment 1	Ipsilateral	416 (124)	22 (2)
		Contralateral	457 (115)	22 (1)
	Segment 2	Ipsilateral	311 (121)‡	37 (9)‡
		Contralateral	323 (111)‡	36 (6)‡
Inferior longitudinal fasciculus	Segment 1	Ipsilateral	604 (153)	29 (1)
		Contralateral	631 (166)	29 (1)
	Segment 2	Ipsilateral	599 (170)	30 (1)
		Contralateral	569 (122)	30 (1)
Uncinate fasciculus	Segment 1	Ipsilateral	356 (154)§	35 (3)§
		Contralateral	436 (150)§	35 (2)§
	Segment 2	Ipsilateral	276 (117)‖	48 (9)‖
		Contralateral	312 (108)‖	47 (5)‖
Fornix	Segment 1	Midline	168 (41)¶	11 (1)¶
	Segment 2	Ipsilateral	160 (62)**	31 (2)**
		Contralateral	173 (56)**	31 (1)**
Corpus callosum Forceps minor	Segment 1	Ipsilateral	950 (289)	34 (2)
		Contralateral	890 (237)	35 (3)

*Patient number 26 excluded from the analysis.
†Patient number 34 excluded from analysis
‡Patient number 6, 8 and 20 excluded from analysis.
§Patient number 32 excluded from the analysis.
‖Patient number 27 excluded from the analysis.
¶Patient number 12 and 14 excluded from analysis.
**Patient number 12 and 25 excluded from analysis.

Supplementary table 2. Full tractography results

Track	n	Cortico-spinal tract	*p*	n	Cortico-spinal tract	*p*	n	Cortico-spinal tract	*p*
DTI scalars		FA ratio - mean (SD)			FA ratio - mean (SD)			FA ratio - mean (SD)	
Segment		1			2			3	
Dislocated tract	8	1.01 (0.05)	0.361	8	1.02 (0.07)	**0.044464**	8	1.04 (0.09)	0.1
Not dislocated tract	26	0.99 (0.05)		26	0.97 (0.06)		26	0.98 (0.08)	
Tumour type AC	18	1.00 (0.05)	0.692	18	0.98 (0.07)	0.617	18	1.00 (0.08)	0.184
Tumor type OD	16	1.00 (0.04)		16	0.99 (0.06)		16	0.98 (0.08)	
Tumour grade II	22	0.99 (0.04)	0.117	22	0.97 (0.06)	0.227	22	0.98 (0.09)	0.126
Tumour grade III	12	1.02 (0.06)		12	1.00 (0.07)		12	1.02 (0.07)	
Tumour side left	18	1.03 (0.04)	**0.000273**	18	1.01 (0.05)	**0.007496**	18	0.99 (0.08)	0.692
Tumour side right	16	0.97 (0.03)		16	0.95 (0.07)		16	1.00 (0.09)	

Track	n	Cortico-spinal tract	*p*	n	Cortico-spinal tract	*p*	n	Cortico-spinal tract	*p*
DTI scalars		MD ratio - mean (SD)			MD ratio - mean (SD)			MD ratio - mean (SD)	
Segment		1			2			3	
Dislocated tract	8	0.99 (0.02)	0.641	8	1.02 (0.02)	0.077	8	1.00 (0.03)	0.529
Not dislocated tract	26	0.99 (0.04)		26	1.01 (0.04)		26	1.02 (0.07)	
Tumour type AC	18	0.98 (0.03)	0.133	18	1.02 (0.03)	**0.007496**	18	1.01 (0.06)	0.931
Tumor type OD	16	1.00 (0.04)		16	1.00 (0.04)		16	1.02 (0.08)	
Tumour grade II	22	0.99 (0.04)	0.843	22	1.01 (0.04)	0.397	22	1.03 (0.08)	0.188
Tumour grade III	12	0.99 (0.04)		12	1.01 (0.02)		12	0.99 (0.02)	
Tumour side left	18	1.00 (0.04)	0.162	18	1.00 (0.04)	0.438	18	1.00 (0.05)	**0.013626**
Tumour side right	16	0.98 (0.04)		16	1.01 (0.03)		16	1.04 (0.08)	

Track	n	Inferior fronto-occipital fasciculus	*p*	n	Inferior fronto-occipital fasciculus	*p*	n	Inferior fronto-occipital fasciculus	*p*
DTI scalars		FA ratio - mean (SD)			FA ratio - mean (SD)			FA ratio - mean (SD)	
Segment		1			2			3	
Dislocated tract	4	1.00 (0.09)	0.28	4	0.99 (0.07)	0.103	4	1.00 (0.09)	0.639
Not dislocated tract	29	0.90 (0.15)		30	0.86 (0.18)		29	0.97 (0.12)	
Tumour type AC	17	0.93 (0.11)	0.46	18	0.88 (0.17)	0.849	18	0.96 (80.11)	0.303
Tumor type OD	16	0.89 (0.17)		16	0.87 (0.18)		15	0.99 (0.12)	
Tumour grade II	22	0.93 (0.12)	0.456	22	0.88 (0.16)	0.843	22	0.98 (0.12)	0.606
Tumour grade III	11	0.87 (0.18)		12	0.87 (0.20)		11	0.96 (0.10)	
Tumour side left	17	0.89 (0.17)	0.627	18	0.86 (0.22)	0.617	17	0.96 (0.14)	0.986
Tumour side right	16	0.93 (0.11)		16	0.89 (0.09)		16	0.98 (0.08)	

Track	n	Inferior fronto-occipital fasciculus	*p*	n	Inferior fronto-occipital fasciculus	*p*	n	Inferior fronto-occipital fasciculus	*p*
DTI scalars		MD ratio - mean (SD)			MD ratio - mean (SD)			MD ratio - mean (SD)	

Segment		1			2			3	
Dislocated tract	4	1.00 (0.03)	0.215	4	1.03 (0.02)	0.979	4	1.03 (0.06)	0.934
Not dislocated tract	29	1.09 (0.18)		30	1.11 (0.21)		29	1.03 (0.08)	
Tumour type AC	17	1.05 (0.08)	0.601	18	1.10 (0.18)	0.309	18	1.04 (0.09)	0.551
Tumor type OD	16	1.12 (0.22)		16	1.10 (0.21)		15	1.01 (0.07)	
Tumour grade II	22	1.05 (0.08)	0.48	22	1.11 (0.21)	0.871	22	1.03 (0.08)	0.456
Tumour grade III	11	1.15 (0.26)		12	1.07 (0.16)		11	1.02 (0.09)	
Tumour side left	17	1.12 (0.22)	0.957	18	1.15 (0.26)	0.986	17	1.04 (0.09)	0.482
Tumour side right	16	1.04 (0.07)		16	1.04 (0.04)		16	1.01 (0.08)	

Track	n	Cingulum	*p*	n	Cingulum	*p*
DTI scalars		FA ratio - mean (SD)			FA ratio - mean (SD)	
Segment		1			2	
Dislocated tract	4	0.89 (0.19)	0.35	4	0.98 (0.08)	0.575
Not dislocated tract	30	0.98 (0.21)		30	0.97 (0.18)	
Tumour type AC	18	0.95 (0.20)	0.501	18	0.94 (0.22)	0.208
Tumor type OD	16	0.99 (0.22)		16	1.02 (0.08)	
Tumour grade II	22	0.95 (0.22)	0.576	22	0.97 (0.20)	0.843
Tumour grade III	12	1.01 (0.18)		12	0.99 (0.12)	
Tumour side left	18	1.13 (0.10)	**0.000002**	18	1.06 (0.06)	**0.000238**
Tumour side right	16	0.80 (0.15)		16	0.88 (0.21)	

Track	n	Cingulum	*p*	n	Cingulum	*p*
DTI scalars		MD ratio - mean (SD)			MD ratio - mean (SD)	
Segment		1			2	
Dislocated tract	4	1.04 (0.03)	0.936	4	1.01 (0.05)	0.769
Not dislocated tract	30	1.05 (0.08)		30	1.07 (0.24)	
Tumour type AC	18	1.05 (0.04)	0.234	18	1.10 (0.31)	1,000
Tumor type OD	16	1.05 (0.11)		16	1.01 (0.04)	
Tumour grade II	22	1.05 (0.09)	0.871	22	1.07 (0.28)	0.358
Tumour grade III	12	1.04 (0.05)		12	1.04 (0.07)	
Tumour side left	18	1.03 (0.04)	0.593	18	1.02 (0.02)	0.479
Tumour side right	16	1.06 (0.11)		16	1.11 (0.33)	

Track	n	Parahippocampal cg	*p*	n	Parahippocampal cg	*p*
DTI scalars		FA ratio - mean (SD)			FA ratio - mean (SD)	
Segment		1			2	
Dislocated tract	1	0.75	1,000	1	0.73	1,000
Not dislocated tract	33	0.93 (0.18)		33	0.92 (0.19)	
Tumour type AC	18	0.90 (0.22)	0.717	18	0.87 (0.23)	0.208

Tumor type OD	16	0.96 (0.11)		16	0.96 (0.11)	
Tumour grade II	22	0.96 (0.14)	0.109	22	0.95 (0.14)	0.256
Tumour grade III	12	0.85 (0.22)		12	0.84 (0.25)	
Tumour side left	18	0.88 (0.18)	**0.0238**	18	0.89 (0.15)	0.184
Tumour side right	16	0.97 (0.16)		16	0.94 (0.23)	
Track	n	Parahippocampal cg	*p*	n	Parahippocampal cg	*p*
DTI scalars		MD ratio - mean (SD)			MD ratio - mean (SD)	
Segment		1			2	
Dislocated tract	1	1.24	1,000	1	1.23	1,000
Not dislocated tract	33	1.09 (0.21)		33	1.06 (0.17)	
Tumour type AC	18	1.13 (0.26)	0.501	18	1.10 (0.22)	0.523
Tumor type OD	16	1.06 (0.12)		16	1.02 (0.09)	
Tumour grade II	22	1.06 (0.14)	0.227	22	1.03 (0.10)	0.256
Tumour grade III	12	1.16 (0.29)		12	1.13 (0.25)	
Tumour side left	18	1.13 (0.25)	0.593	18	1.09 (0.19)	0.051
Tumour side right	16	1.05 (0.14)		16	1.03 (0.15)	
Track	n	Arcuate fasciculus	*p*	n	Arcuate fasciculus	*p*
DTI scalars		FA ratio - mean (SD)			FA ratio - mean (SD)	
Segment		1			2	
Dislocated tract	4	0.99 (0.08)	0.229	3	0.99 (0.08)	0.57
Not dislocated tract	30	0.89 (0.15)		28	0.91 (0.15)	
Tumour type AC	18	0.88 (0.16)	0.361	16	0.86 (0.16)	**0.03445**
Tumor type OD	16	0.93 (0.13)		15	0.99 (0.08)	
Tumour grade II	22	0.90 (0.15)	0.9	21	0.91 (0.14)	0.657
Tumour grade III	12	0.91 (0.15)		10	0.94 (0.15)	
Tumour side left	18	0.95 (0.11)	0.081	16	1.00 (0.12)	**0.000347**
Tumour side right	16	0.85 (0.16)		15	0.84 (0.12)	
Track	n	Arcuate fasciculus	*p*	n	Arcuate fasciculus	*p*
DTI scalars		MD ratio - mean (SD)			MD ratio - mean (SD)	
Segment		1			2	
Dislocated tract	4	1.00 (0.03)	0.323	3	1.00 (0.02)	0.404
Not dislocated tract	30	1.14 (0.24)		28	1.08 (0.16)	
Tumour type AC	18	1.19 (0.27)	**0.008303**	16	1.14 (0.19)	**0.003231**
Tumor type OD	16	1.05 (0.15)		15	1.01 (0.04)	
Tumour grade II	22	1.10 (0.17)	0.871	21	1.07 (0.15)	0.434
Tumour grade III	12	1.16 (0.32)		10	1.09 (0.17)	
Tumour side left	18	1.05 (0.15)	**0.01237**	16	1.05 (0.14)	0.333

Tumour side right	16	1.21 (0.28)		15	1.10 (0.17)	
Track	n	Inferior longitudinal fasciculus	*p*	n	Inferior longitudinal fasciculus	*p*
DTI scalars		FA ratio - mean (SD)			FA ratio - mean (SD)	
Segment		1			2	
Dislocated tract	3	0.95 (0.09)	0.903	3	0.97 (0.06)	1,000
Not dislocated tract	31	0.93 (0.15)		31	0.96 (0.10)	
Tumour type AC	18	0.89 (0.16)	**0.026**	18	0.93 (0.11)	0.076
Tumor type OD	16	0.99 (0.11)		16	0.99 (0.08)	
Tumour grade II	22	0.95 (0.13)	0.46	22	0.97 (0.10)	0.652
Tumour grade III	12	0.90 (0.17)		12	0.95 (0.10)	
Tumour side left	18	0.93 (0.15)	0.769	18	0.93 (0.11)	**0.028**
Tumour side right	16	0.94 (0.14)		16	1.00 (0.08)	
Track	n	Inferior longitudinal fasciculus	*p*	n	Inferior longitudinal fasciculus	*p*
DTI scalars		MD ratio - mean (SD)			MD ratio - mean (SD)	
Segment		1			2	
Dislocated tract	3	1.04 (0.09)	0.808	3	1.03 (0.08)	0.808
Not dislocated tract	31	1.05 (0.11)		31	1.02 (0.09)	
Tumour type AC	18	1.08 (0.14)	0.056	18	1.04 (0.11)	0.343
Tumor type OD	16	1.01 (0.06)		16	1.00 (0.06)	
Tumour grade II	22	1.04 (0.10)	0.552	22	1.02 (0.09)	0.652
Tumour grade III	12	1.07 (0.14)		12	1.04 (0.09)	
Tumour side left	18	1.06 (0.11)	0.617	18	1.05 (0.10)	0.162
Tumour side right	16	1.04 (0.11)		16	1.00 (0.07)	
Track	n	Uncinate fasciculus	*p*	n	Uncinate fasciculus	*p*
DTI scalars		FA ratio - mean (SD)			FA ratio - mean (SD)	
Segment		1			2	
Dislocated tract	0	NA*	NA*	0	NA*	NA*
Not dislocated tract	33	0.91 (0.15)		33	0.91 (0.17)	
Tumour type AC	18	0.92 (0.13)	1,000	18	0.91 (0.17)	0.899
Tumor type OD	15	0.91 (0.17)		15	0.92 (0.18)	
Tumour grade II	22	0.93 (0.14)	0.633	21	0.93 (0.16)	0.443
Tumour grade III	11	0.90 (0.16)		12	0.88 (0.18)	
Tumour side left	17	0.88 (0.15)	0.358	17	0.89 (0.21)	0.928
Tumour side right	16	0.95 (0.13)		16	0.94 (0.11)	
Track	n	Uncinate fasciculus	*p*	n	Uncinate fasciculus	*p*
DTI scalars		MD ratio - mean (SD)			MD ratio - mean (SD)	

Segment		1			2	
Dislocated tract	0	NA*	NA*	0	NA*	NA*
Not dislocated tract	33	1.07 (0.12)		33	1.10 (0.20)	
Tumour type AC	18	1.06 (0.10)	0.551	18	1.11 (0.21)	0.416
Tumor type OD	15	1.09 (0.15)		15	1.08 (0.19)	
Tumour grade II	22	1.06 (0.09)	0.985	21	1.10 (0.22)	0.808
Tumour grade III	11	1.10 (0.17)		12	1.09 (0.16)	
Tumour side left	17	1.09 (0.15)	0.787	17	1.17 (0.24)	**0.0244**
Tumour side right	16	1.05 (0.07)		16	1.02 (0.10)	
Track	n	Fornix	*p*	n	Fornix	*p*
DTI scalars		FA ratio			FA ratio mean (SD)	
Segment		1 - midline			2	
Dislocated tract	NA†	NA†	NA†	8	NA*	NA*
Not dislocated tract	NA†	NA†		26	NA*	
Tumour type AC	NA†	NA†	NA†	17	1.02 (0.11)	0.521
Tumor type OD	NA†	NA†		15	1.01 (0.08)	
Tumour grade II	NA†	NA†	NA†	21	1.02 (0.08)	0.552
Tumour grade III	NA†	NA†		11	1.00 (0.11)	
Tumour side left	NA†	NA†	NA†	17	1.03 (0.09)	0.497
Tumour side right	NA†	NA†		15	1.00 (0.09)	
Track	n	Fornix	*p*	n	Fornix	*p*
DTI scalars		MD ratio			MD ratio - mean (SD)	
Segment		1 - midline			2	
Dislocated tract	NA†	NA†	NA†	8	NA*	NA*
Not dislocated tract	NA†	NA†		26	NA*	
Tumour type AC	NA†	NA†	NA†	17	1.04 (0.18)	0.91
Tumor type OD	NA†	NA†		15	1.03 (0.20)	
Tumour grade II	NA†	NA†	NA†	21	1.06 (0.19)	0.19
Tumour grade III	NA†	NA†		11	0.98 (0.17)	
Tumour side left	NA†	NA†	NA†	17	0.95 (0.12)	**0.011403**
Tumour side right	NA†	NA†		15	1.12 (0.20)	
Track	n	Callosal body minor forceps	*p*			
DTI scalars		FA ratio - mean (SD)				
Segment		1				
Dislocated tract	0	NA*	NA*			
Not dislocated tract	34	0.97 (0.09)				
Tumour type AC	18	0.99 (0.06)	0.501			

Tumor type OD	16	0.95 (0.12)	
Tumour grade II	22	0.97 (0.09)	0.928
Tumour grade III	12	0.97 (0.09)	
Tumour side left	18	1.00 (0.07)	0.133
Tumour side right	16	0.94 (0.10)	

Track	n	Callosal body minor forceps	*p*
DTI scalars		MD ratio - mean (SD)	
Segment		1	
Dislocated tract	0	NA*	NA*
Not dislocated tract	34	1.04 (0.07)	
Tumour type AC	18	1.01 (0.03)	0.133
Tumor type OD	16	1.07 (0.09)	
Tumour grade II	22	1.04 (0.07)	0.601
Tumour grade III	12	1.05 (0.08)	
Tumour side left	18	1.04 (0.07)	0.309
Tumour side right	16	1.04 (0.07)	

*No dislocated tracts

†No ratio due to tract segment in midline

FA and MD ratios were compared between groups (FA ratio= ipsilateral mean FA divided with contralateral mean FA, MD ratio = ipsilateral mean MD divided with contralateral mean MD). Mann-whitney U tests. $p<0.05$ was regarded as statistically significant

FA = fractional anisotropy, MD = mean diffusivity, cg = cingulum
